# Development of solid lipid nanoparticles-loaded drugs in parasitic diseases

**DOI:** 10.1186/s11671-023-03955-w

**Published:** 2024-01-04

**Authors:** Sara Nemati, Mahsa Mottaghi, Parisa Karami, Hamed Mirjalali

**Affiliations:** 1 Foodborne and Waterborne Diseases Research Center, Research Institute for Gastroenterology and Liver Diseases, Shahid Beheshti University of Medical Sciences, Tehran, Iran; 2 Department of Biology, Faculty of Basic Sciences, Science and Research Branch, Islamic Azad University, Tehran, Iran

**Keywords:** Nano-carriers, Drug delivery, Solid lipid nanoparticles, Protozoa, Helminths

## Abstract

Parasites cause illnesses with broad spectrum of symptoms from mild to severe, and are responsible for a significant number of outbreaks in the world. Current anti-parasitic drugs are toxic and have significant side effects. Nano-carriers are believed to obviate the limitations of conventional drugs via decreasing side effects and increasing target delivery and drug permeability with a controlled prolonged release of a drug. Solid lipid nanoparticles (SLNs) are lipid nanoparticles (LNPs), which have frequently been practiced. Suitable release rate, stability, and target delivery make SLNs a good alternative for colloidal carriers. SLNs are supposed to have great potential to deliver natural products with anti-parasitic properties. Nanoparticles have employed to improve stability and capacity loading of SLNs, during recent years. This review describes development of SLNs, the methods of preparation, characterization, and loaded drugs into SLNs in parasitic diseases. In addition, we summarize recent development in anti-parasitic SLNs-loaded drugs.

## Introduction

Nanotechnology is a wide study area, which covers broad range of fields such as food, medicine, electronics, computer, communication, transportation, energy, and environment [1–3]. Advanced progress in nanotechnology has been led to control and use of materials at a nano-metric dimensions [4]. Actually, the main feature of nano materials is their dimension, which is smaller than 100 nm [4]. The main application of nanotechnology in biomedicine is designing and developing nano-carriers to sufficiently and specifically deliver and release therapeutic agents [5, 6]. Actually, nanomedicine employs nanotechnology, biomedical, and pharmaceutical sciences for either diagnosis or delivery purposes [7, 8] to increase and decrease effectiveness and toxicity, respectively [9, 10].

Colloidal particles are solid nano-carriers, composed from synthetic and natural polymers with sizes between 10 and 1000 nm, which could be an alternative for liposomes colloidal carriers [11]. Most of the therapeutic agents are conjugated to nanoparticles (NPs) to alter pharmacokinetic (PK) and/or pharmacodynamics (PD) properties of drugs [6, 12, 13]. Furthermore, many nano-drug formulations have been approved in clinical trials, so far [14, 15]. The types of NPs in approved and investigational drugs are polymeric, liposomal, nanocrystal formulations, inorganic NPs, micelles, metals/metal oxides, proteins, and dendrimers [16–18]. The majority of employed NPs, which have already been proven, are liposomes and polymers [14, 17]. Liposomes are small solid particles, suspended in a fluid phase, with a significant role in smart drug delivery with excellent efficiency [19]. The successful delivery of drugs depends on the sustained release of their contents and their stability in nanometer size [20, 21]. Due to the size, safety, ability to encapsulate various drugs, biocompatibility, and excellent modulation, liposomal colloidal drug carriers are good alternatives as a drug transporter compared to the expensive polymers [22]. Solid lipid nanoparticles (SLNs) are member of lipid nanoparticles, which due to their various therapeutic applications, are alternative carriers for traditional colloidal nanoparticles.

Parasites including protozoa and helminths, are widely reported in the world, and are responsible for gastrointestinal disorders, malnutrition, anemia, allergies, and etc. The main transmission routes of parasites are ingestion of contaminated food and water or through vectors (Table 1) [23–25]. Efficient treatment is a critical challenge facing with a broad range of medical and veterinary important parasites. The chemical drugs used for parasitic diseases are mostly expensive and toxic, with side effects and drug resistance [26–28]. Therefore, developing new efficient drugs for treatment of parasitic diseases has been practiced. Accordingly, nanotechnology has been incorporated in pharmaceutics to develop effectual drug delivery system for various parasitic diseases such as toxoplasmosis [29, 30], leishmaniasis [31–36], malaria [37–40], and trypanosomiasis [41]. Encapsulation of conventional drugs into nano-carriers such as lipid nanoparticles provides the possibility to develop new drugs with higher efficiency than common drugs with a low toxicity to host cells. In this review the general aspects, preparation methods, and characterization techniques of SLNs are explored. The current herbal and chemical antiparasitic agents are summarized and their combination with SLNs are reviewed. In addition, we comprehensively discuss the current development of nanodrugs for parasitic diseases.

**Table 1:** A summary of medical important parasites and the relevant diseases

Parasites		Genera/species	Diseases
Protozoa	Gastrointestinal	*Entamoeba histolytica*	Amebiasis
		*Giardia lambelia*	Giardiasis
		*Cryptosporidium* spp*.*	Cryptosporidiosis
		*Isospora* spp*.*	Isosporiasis
		*Sarcocystis* spp*.*	Sarcocystosis
	Urinary tract	*Trichomonas vaginalis*	Trichomoniasis
	Blood and tissue	*Plasmodium* spp*.*	Malaria
		*Leishmania* spp*.*	Cutaneous leishmaniasis, visceral leishmaniasis, mucocutaneous leishmaniasis
		*Trypanosoma* spp*.*	African sleeping sickness, Chagas
		*Toxoplasma gondii*	Toxoplasmosis
	Free living amoebae (FLA)	*Naegleria* spp. *Acanthamoeba* spp.	Primary amebic meningoencephalitis (PAM), *Acanthamoeba* keratitis (AK), Granulomatous amebic encephalitis (GAE)
Helminths	Nematoda	*Ascaris* spp.	Ascariasis
		*Enterobius* spp.	Enterobiasis
		Hookworms	Ancylostomiasis Necatoriasis
		*Strongyloides* spp.	Strongyloidiasis
		*Trichostrongylus* spp.	Trichostrongylosis
		*Trichuris* spp.	Trichuriasis
		*Trichinella* spp.	Trichinosis
		*Toxocara* spp.	Toxocariasis
		*Filaria* spp.	Lymphatic filariasis, River blindness
	Cestoda	*Diphyllobothrium* spp.	Diphyllobothriasis
		*Hymenolepis* spp.	Hymenolepiasis
		*Taenia* spp. *Taenia solium* (pork tapeworm) *Taenia saginata* (beef tapeworm)	*Taeniasis,* Cysticercosis (*T. solium*)
		*Echinococcus* spp. *E. granulosus* *E. multilocularis* *E. vogeli*	Echinococcosis Cystic echinococcosis (*E. granulosus)* Alveolar echinococcosis (*E. multilocularis)* Polycystic echinococcosis (*E. vogeli*)
	Trematoda	*Fasciola hepatica*	Fascioliasis
		*Echinostoma ilocanum*	Echinostomiasis
		*Schistosoma* spp.	Schistosomiasis
		*Clonorchis sinensis*	Clonorchiasis
		*Paragonimus westermani*	Paragonimiasis

## General aspects of SLNs (structure, application, and characterization)

**Fig. 1:**
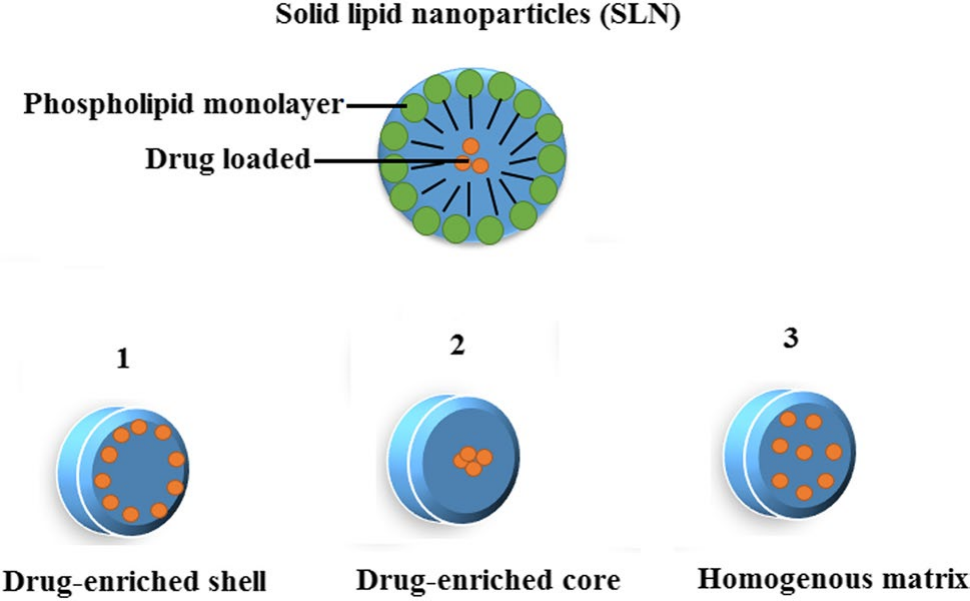
Structure of SLNs models including 1: Drug-enriched shell model, 2: Drug-enriched core model, 3: Homogenous matrix model

In last decades, several colloidal carrier systems such as emulsions, liposomes and polymeric micro, and nanoparticles have been developed [42–44]. Among carrier systems, SLNs as an alternative carrier system to traditional colloidal carriers, are attracting major attention for various therapeutic applications [42, 45]. At the early 90’s decade, SLNs were offered as an alternative carrier to colloidal drug carriers [46]. The size of SLNs as a sub-micron colloidal vehicle, are ranging from 50 to 1000 nm [44]. The large surface area, higher stability, and high drug loading properties, which improve efficacy of pharmaceuticals, are the most important advantages of SLNs [47]. In addition, SLNs have better performance in controlling the release kinetics compared to liquid lipid, which makes them more beneficial for intravenous applications [48]. The lipid matrix structure of solids (which is made of physiological lipids) reduces toxicity of SLNs and improves the absorption of water-insoluble drugs in the intestine [49, 50]. SLNs are comprised of a solid core of a high melting point lipid surrounded by phospholipids as a layer of safer surfactant [51, 52]. The lipids in SLNs include: (1) partial glycerides (2) saturated monoacid triglycerides (3) fatty acids, and (4) waxes. The toxicity profiling of SLNs is important for both production and application of such carrier system. According to the encapsulation location of drug compounds, SLNs have three different structures, which are categorized based on their preparation methods: (1) Drug-enriched shell model, in which NPs are prepared by a hot homogenization process. (2) Drug-enriched core model, which leads to supersaturation of the active drug based on cooling the nanoemulsion methods, and (3) homogenous matrix model that NPs are prepared by the cold homogenization process [53] (Fig. 1).

The toxicity profile is important for the generation and application of SLN systems [54, 55]. Toxicological profile should be evaluated before pre-clinical and clinical studies using in vitro and in vivo assays [56].

**Fig. 2:**
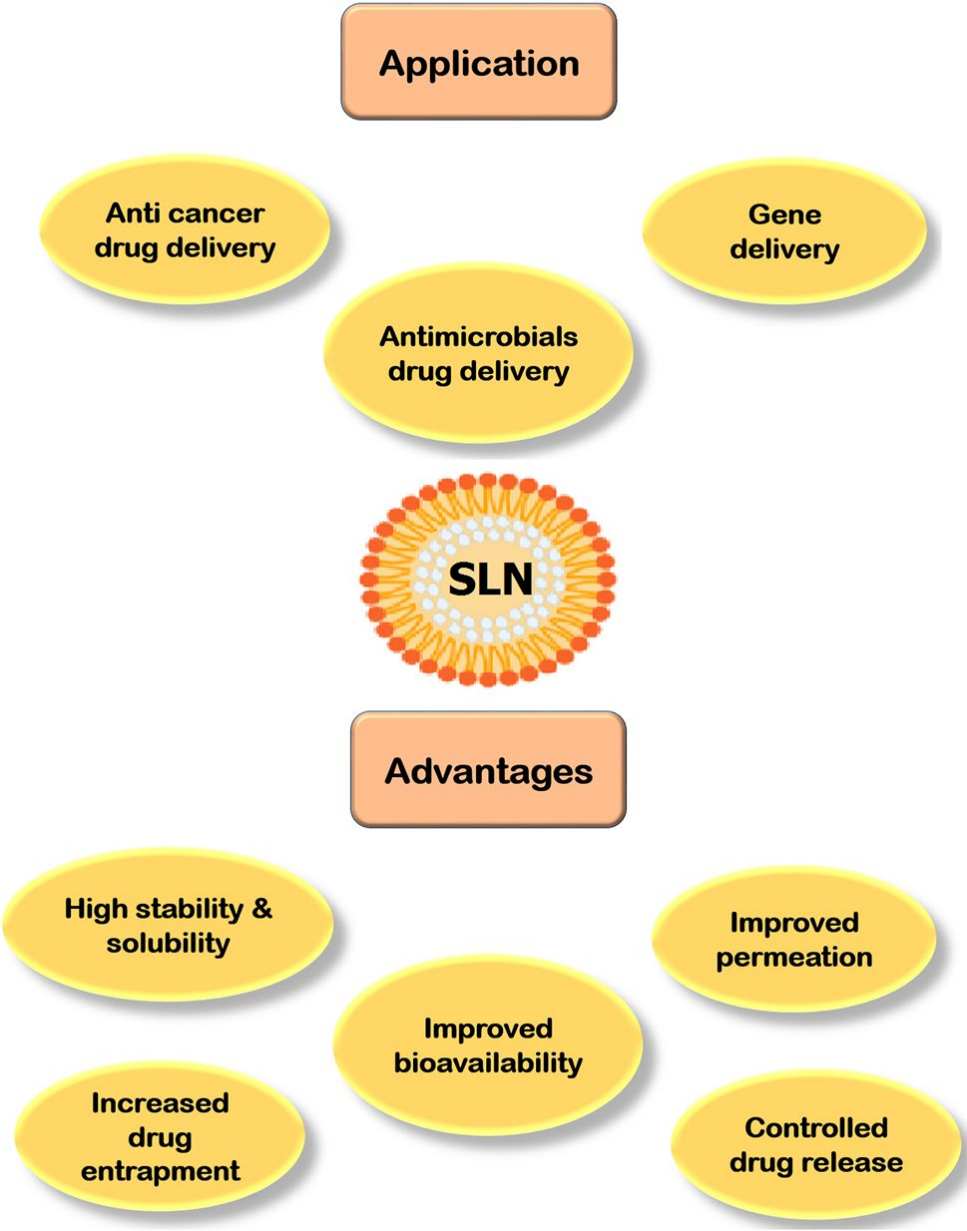
Applications and advantages of SLNs are schematically depicted

It has been reported that SLNs could be a target carrier for variety of drugs [57, 58]. The advantages of SLNs such as stability, biodegradable, and small size suggest them capable for delivering drugs to the liver [59]. Actually, because of the high saturation solubility and rapid dissolution rate, SLNs can accelerate the early stages of drug action [53] (Fig. 2).

Recent research has shown various potential administrations of SLNs such as oral route, brain, parenteral, ophthalmic, topical, and transdermal delivery, and carrying gene vector [49, 59–62]. The other advantage of SLNs is the modulation of drug release profiles [42]. Drug release from SLNs depends on the factors such as particle size, surfactant concentration, polymorphic state of SLNs, and temperature [48, 63]. Solid matrix of SLNs contains well-tolerated ingredients that protect active ingredients against chemical degradation. In addition, due to the initially burst of released drug, SLNs can prolong drug release time and minimize the burst due to increasing the solubility of the drug in the water phase, temperature, surfactant, and concentration [64, 65].

A variety of methods have been developed for characterization of SLNs. Evaluation of particle size, crystallinity size, distribution kinetics (zeta potential), lipid polymorphism, coexistence of micelles liposome, drug nanoparticles shape, drug release, and surface morphology are measured using various methods such as transmission electron microscopy (TEM), scanning electron microscopy (SEM) [66], scanning tunneling microscopy (STM), freeze fracture electron microscopy (FFEM), atomic force microscopy (AFM), photon correlation spectroscopy (PCS), and dynamic light scattering (DLS) [67]. In addition, the entrapped therapeutic agent volume can be characterized by UV, visible spectrophotometer, and high-pressure liquid chromatography (HPLC) [68].

The location of the therapeutic agent in the SLNs can be effective on its release, as well. For instance, the rate of drug release, which is located in the shell of the SLNs, is faster than the therapeutic agent that is located in the lipid nucleus. The properties and efficacy of SLNs were evaluated by in vivo and ex vivo assays, such as evaluation of drug loading and entrapment efficiency (EE) [69], assessment of the percentage of drug release [70], pharmacokinetic studies [71], determination of particle size [72], animal studies [73], evaluation of polydispersity index (PDI) [74], zeta potential (ZP) [75], thermogravimetric analysis (TG) [76], and cell toxicity by methyl-thiazolyl-tetrazolium (MTT) colorimetric assay [77].

## Preparation of SLNs

Many techniques are available for the preparation of SLNs [78, 79]. The suitable preparation technique is chosen based on the particle size and availability of technique, which improve drug loading and successful encapsulation of therapeutic agent. SLNs are prepared from lipid, emulsifier, and water/solvent. The preparation of SLNs requires a precursor such as emulsion, micro-emulsions, and micellar solutions. The most common techniques employed for emulsions are hot homogenization, melt dispersion [80], phase inversion temperature (PIT) [81], and solvent evaporation-diffusion from emulsions [82]. Microemulsion dilution and microemulsion cooling techniques are common techniques for microemulsions [83], while coacervation method [84] is common in micellar solutions. Some of other techniques such as membrane contactor technique [85], spray-drying, spray-congealing [86] and electrospray are based on a particular instrument. However, high shear homogenization, hot and cold homogenization, ultra-sonication or homogenization, emulsification/evaporation, microemulsion, double emulsion method, and solvent evaporation/diffusion from emulsions are reported as the most common preparation techniques of SLNs formulation for anti-parasitic drugs [87–89].

Apart from conventional preparation methods, recent innovated technologies are described for preparation of liposomes, such as freeze drying of monophase solutions method, microfluidic channel method, membrane contactor method, dense gas techniques, green technology [90]. Among the new methods, green technologies have many benefits in biomedical research. Actually, the process of development of synthetic nanomaterials could be expensive, with less feasibility, which can produce environmental harmful substances [91]. In this regard, applications of green technologies had been led to 7% decrease in production of toxic materials such as hydrochloric acid (Hcl), trichloroethylene, and methyl isobutyl ketone, only during a decade from 2003 to 2014 [92]. Green technologies have been incorporated to synthesize lipid nanoparticles. Tocosome, a novel drug delivery system, which was firstly described by Mozafari et al. [93], is a bioactive carrier. This carrier is a product of a modified and improved heating method, which is known as “Mozafari method” [94]. Although green technologies have been tried for some parasitic diseases such as malaria [95, 96], the application of green technologies to prepare lipid NPs for parasitic diseases are rare and needs further consideration (Table 2).

**Table 2:** Preparation methods of SLNs and their advantages and disadvantages

Methods	Advantages	Disadvantages	Parasites	References
High shear homogenization (HSH)	1. Production of solid lipid nano-dispersions 2. wide-spreading and easy to handle	1. Degradation of powder, especially for fragile or thermolabile powders 2. Over wetting cause the generation of large lumps. Produces less compressible granules	*S. mansoni* *P. falciparum* *L. tropica* *L. major* *E. granulosus* *T. spiralis*	[31, 34, 35, 97–100]
Hot homogenization	The production of colloidal suspensions of SLN without solvents	1. Drug expulsion from nanoparticles 2. No encapsulation of hydrophilic drugs 3. Not suitable for thermo-sensitive drugs 4. Low drug loading in the SLN	*S. mansoni*	[101]
Cold homogenization	1. Dealing with various problems associated with hot homogenization 2. Larger particle sizes and a broader size distribution	1. Energy intensive process 2. Polydisperse distributions 3. Unproven scalability	*P. falciparum*	[40]
Ultrasonication/Homogenization	Common in every lab	Broader particle size distribution ranging into micrometer range	*E.* *granulosus*	[102]
Emulsification/Evaporation	Avoidance of any heat	Toxicological problems arising from solvent residues	*L. donovani*	[36]
Microemulsion templates (a) Microemulsion dilution technique (b) Microemulsion cooling technique	1. Microemulsions form upon simple mixing of the components 2. No need for high input energy 3. Advantageous drug loading within SLN 4. Simple, reproducible, easy to scale-up 5. Biocompatibility 6. Cost-effectiveness	Their structure cannot be observed through an optical microscope	*E. granulosus* *S. mansoni*	[98–100]
Supercritical fluid	1. High solvent extraction efficiency using supercritical CO_2_ 2. Highly-compressed fluid that combines the properties of gases and liquids	Very limited applications for lipid particles	–	–*
Double emulsion method	1. Simple 2. Appropriate in controlling process parameters 3. Encapsulating both lipophilic and hydrophilic drug molecules	Large particles are obtained with this technique	*H. contortus* *Leishmania* spp. *T. gondii*	[30, 31, 33, 103]
Spray-drying	1. Ability to manipulate and control a variety of parameters 2. The choice method of drying of many thermally-sensitive materials	1. May have convoluted surfaces, asperities and cavities 2. The produced particles are not always spherical	–	–*
Coacervation method	1. Economical for laboratory and industrial application 2. Possibility to control the size of SLN 3. Production of microspheres and microcapsules	Particle size is highly influenced by the lipid concentration	–	–*
Solvent based methods	1. Encapsulate molecules with stability and bioavailability problems 2. Mild operating temperature	–	–	–*
Solvent injection method	1. Efficient 2. Versatile 3. Simple 4. Applicable to both hydrophobic and hydrophilic drugs	Requires organic solvents and is not easily scaled up	*T. gondii*	[29]
Solvent evaporation – diffusion from emulsions	Solvent diffusion is more innovative and most of the solvent employed show a better safety profile compared to volatile solvents	1. Solvent evaporation technique is quite obsolete with many drawbacks 2. Enquire organic solvents 3. Enquire high surfactant levels 4. Complicated 5. Enquire long processes	*E. granulosus* *L. donovani*	[36, 104]
Phase inversion temperature method	1. Easy to scale up 2. Use less energy	Very limited applications for thermosensitive molecules ( lipid, proteins)	*T. canis*	[105]
Electrospray	1. A major impact on the mass spectrometric analyses 2. High sensitivity 3. Selectivity of analyte detection 4. High molecular weight polymers can be used 5. Low cost 6. Uses fewer solvents 7. Not use surfactant 8. Produce little residue	1. Small amount of particles is obtained 2. In some cases uses a cross-linking agent	–	–*
Membrane contactor technique	1. Simple 2. Fast 3. Potential for an industrial scale-up 4. Production of nanosized liposome suspensions on a large scale	–	–	–*
Green technologies	1. Simple 2. Not expensive 3. The use of non-toxic materials 4. Low maintenance 5. High sustainability	1. The synthesis conditions need to be carefully optimized 2. Control of morphology and crystallinity		[95, 96]
Mozafari method	1. Employing green technologies 2. Economical 3. Storage stability 4. Simple	Not reported yet	–	–*

^*^There is no study on parasites

## Herbal and natural polymer components loaded into SLNs

Herbal medicine has been developed as a remedy for diseases along with human evolution [106, 107]. Over the time, because of the advantages of herbal medicines, applications of this approach have been developed [7, 108]. Currently, herbal compounds are incorporated with a nanostructured system to improve molecular size, increase bioavailability and biocompatibility, and decrease probable toxicity of the compounds [10, 109–111]. Indeed, nanotechnology can reduce side effects and increase target delivery of the herbal products [112–114]. In recent years, SLNs have taken attention by the researchers in drug delivery area [115] to enhance the efficacy [116–118] and improve oral bioavailability [119] of conventional herbal medicine. Furthermore, SLNs are most stable vehicles for plant extracts, which have shown higher antioxidant activity [120].

Artemisinin, an extract of the *Artemisia annua,* is one of the well-known herbal extract in medicine, which has been prescribed for malaria treatment for a long time. To increase efficiency and decrease side effects, artemisinin-based combination therapies (ACTs) have recently been developed for treatment of malaria. Many studies have employed SLNs formulation as a carrier for antimalarial drugs such as artemisinin [121]. In this regard, artemisinin and its derivatives (artesunate, artemether, dihydroartemisinin) are combined with other commercial drugs [122, 123]. Currently, combination therapy is preferred over monotherapy for malaria treatment [124]. For example, Attama et al. [38] employed SLNs to encapsulate two antimalarial drugs, artemether and lumefantrine, as the first-line treatment for malaria. In this regard, artemether and lumefantrine were loaded into the lipid nanoparticle to decrease side effects, enhance bioavailability, and overcome the pharmacokinetic mismatches. In addition, SLNs were labeled with coumarin 6 to indicate cellular uptake of encapsulated antimalarial drugs in *Plasmodium*-infected cells. The results of in vivo tests revealed high parasitemia clearance after oral administration with lower side effect, suggesting SLNs formulation as a promising technique to increase efficiency of combination therapy for malaria. Dwivedi et al. [39], loaded another derivations of artemisinin, arteether (ART), into SLNs for oral prescribtion. To evaluate the efficacy of ART-SLNs formulation, the entrapment efficiency was calculated using high-performance liquid chromatography (HPLC) method. The cytotoxicity effects of ART were evaluated out using MTT assay on J774A.1 cell line that the results showed a cell viability over 90%. Omwoyo et al. [37], loaded dihydroartemisinin (DHA), a derivative of artemisinin, into SLNs to evaluate anti-malaria efficacy, and to overcome limitations of this component including poor water-solubility and poor pharmacokinetic profile. The SLNs-loaded DHA was prepared based on single-emulsion solvent evaporation and high-speed homogenization methods, with size range of range from 150 to 500 nm. Stability and sustained release of drug were up to 90 days and over 20 h, respectively. Moreover, IC_50_ was 0.25 ng/ml with 97.24% chemo-suppression at 2 mg/kg/day, which were evaluated by in vitro and in vivo assays, respectively. Eventually, the released results using in vitro and in vivo assays showed that SLNs formulation was excellent for clinical practices.

Recently, numerous reports represented a ressistance agaist artemisinin [125–128]. Luteolin as a bioactive component can be utilized to eradicate artemisinin-resistant *P. falciparum* [129]. Luteolin is able to disrupt the life cycle of the parasite by preventing the fatty acid biosynthesis, which prevents the formation of new organelles and development of young trophozoite (ring stage) [130, 131]. Because of excellent biocompatibility of luteolin, this compound has been loaded into SLN-polyethylene glycol (PEG) using heat homogenization, cold homogenization, and hot-microemulsion ultrasonic methods [40]. Accordingly, luteolin encapsulated by SLN with further PEG modification showed an improvement of relative bioavailability, with decreased distribution and clearance of the component.

*T. gondii* is another prevalent human-infecting protozoa, which is the considered as a target for development of new treatment. Nemati et al. [30] developed neem oil-SLNs (NeO-SLNs) using double emulsification method and evaluated its anti-*Toxoplasma* activity. The results of this study showed that SLNs, as a liposomal carrier for NeO, prolonged extract release with an acceptable anti-*Toxoplasma* activity and cell cytotoxicity.

Chitosan (CS) is a natural biopolymer material composed of *N*-acetyl-d-glucosamine and d-glucosamine units with interesting properties and numerous pharmaceutical applications, which has been widely employed in the drug delivery area [132, 133]. In recent years, novel formulation against microbial agents with natural origins and nontoxic compounds have attracted the attention of many researchers [134]. The CS is a new delivery system, which has been widely studied as a coating material for different nanoparticles including SLNs [135, 136]. CS -coated SLN was successfully formulated to treat several diseases [22, 137, 138]. The anti-effects CS against *Leishmania*, *Trichomonas*, *Plasmodium* and *Toxoplasma* has previously been reported [139]. In this regard, Teimouri et al. [140] showed that CS was highly effective against *T. gondii*, and in a nanoformulation can be used as an alternative natural medicine in the treatment of toxoplasmosis. The results of in vitro and in vivo experiments showed 100% mortality of tachyzoites of RH strain and growth inhibition rates of tachyzoites in peritoneal of mice. CS exhibited significant effects on *P. berghei*, as well [141]. The results of in vivo and in vitro experiments showed that different concentrations of chitosan have potential antimalarial activity. To overcome the efflux of chloroquine (CQ) from the parasite's acidic digestive vacuole, nanoparticle chloroquine (NCQ) was employed. CS encapsulated nanochloroquine attenuated *P. berghei* infection in male Swiss mice, while the nanoformulation showed more potency to protect DNA damage, oxidative stress, and inflammation in infected mice [141]. The antiparasitic activity of chitosan was also highlighted on *T. gallinae* trophozoites. Accordingly, CS concentrations showed high mortality rate in treated trophozoites compared to control group. In addition, CS inhibited the viability of *T. gallinae* trophozoites [142, 143]. However, few studies have been conducted on combination of CS with SLN in parasitic infections. Additionally, several studies have been shown that CS has in vitro antileishmanial activity with 50% (EC_50_) against promastigotes and amastigotes forms of *L. infantum*, *L. amazonensis*, *L. chagasi*, *L. major, L. tropica,* and *L. mexicana* [144–147]. Moreover, recent findings reported that chitosan and its derivatives represented therapeutic and vaccine purposes in the treatment and prevention of both cutaneous and visceral leishmaniasis. Therefore, commercial chitosan is suggested to be an appropriate candidate for further studies on treatment of cutaneous and visceral leishmaniasis. In a study by Jain, et al. [36], the CS-coated SLNs was employed as an immunoadjuvant chemotherapy for *Leishmania* infection. The combination of SLNs with CS, as natural polymer, loaded with amphotericin B (AmB), induced activation of macrophages, which provoked immune responses such as tumor necrosis factor (TNF) α and interleukin (IL) 12 against leishmaniasis. SLNs preparation was performed using solvent emulsification-evaporation method and the cytotoxic studies in mice revealed suitable safety profile. The result indicated that AmB-SLNs as a safe and effective drug delivery system can be useful in anti-*Leishmania* therapy and immunotherapy field.

## The therapeutic efficacy of antiparasitic drugs loaded into SLNs

In order to improve the therapeutic properties and increase the effectiveness of commercial antiparasitic drugs, many studies have applied SLNs as suitable carriers to transfer therapeutic agents, such as praziquantel [98, 101], paromomycin (PM) [29, 32, 34, 35], nitazoxanide (NTZ) [148], tanespimycin (17-AAG) [31], and AmB [32, 36, 149] in parasitic diseases.

### Protozoa

Available data indicates side effects, toxicity, and resistant to commercial drugs in different parasitic protozan infections [150, 151]. To overcome the limitations of available chemical drugs for parasitic protozoan infections, the lipid-based formulations were suggested to enhance drug bioavailability and efficacy [152, 153]. Parvez et al. [32], developed and designed a drug-carrier system to decrease drug toxicity and improve bioavailability of AmB and PM against visceral leishmaniasis (VL), as orally administered dual drug SLNs (DDSLNs). For this purpose, the SLNs were modified with a dextrin composition, 2-hydroxypropyl-*β*-cyclodextrin (HPCD), which was incorporated with AmB and PM. The in vitro and in vivo tests were performed in mouse macrophage cells (J774) and *L. donovani*-infected BALB/c mice, respectively. As a results, DDSLNs decreased drug toxicity and side effects and enhanced the efficacy, compared to liposomal forms. In addition, the HPCD modification improved the uptake of SLNs by the infected macrophages and inhibited intracellular amastigote growth [32]. Moreover, vitamin B_12_-stearic acid (VBS), which was coated with AmB-loaded SLNs (VBS-AmB-SLNs), were prepared by double emulsion, solvent evaporation, and thermal-sensitive hydrogel methods to improve bioavailability for oral delivery and enhance the absorption potency of AmB [33]. Accordingly, the results of anti-leishmanial activity and cellular uptake revealed an enhanced efficacy up to 94% with the low toxicity percentage on cell lines. Actually, the most desirable rout to absorb drugs in the drug delivery system is the oral delivery, but the oral absorption of AmB is 0.2–0.9%, and nanoformulations can increase absorption and efficacy of the drug [33].

It was suggested that modulation of heat shock protein 90 (Hsp90) can inhibit the growth of *Leishmania* spp [154]. Actually, it was reported that 17-N-allylamino-17-demethoxygeldanamycin (17-AAG; tanespimycin) is an hsp90 inhibitor [155]. Pires et al. [31] developed a novel generation of anti-*Leishmania* agents by loading SLNs on tanespimycin (17-AAG, tanespimycin) using a double emulsion method with a particle size of about 104.3 ± 1.2 to improve the therapeutic effects of the drug. The findings showed a remarkable drug internalization, which seems to be potentially important delivery systems for elimination of intracellular *Leishmania* [154]. However, some drawbacks like the poor aqueous solubility and low stability have been limited usage of tanespimycin. Kharaji, et al. [34] developed a novel delivery systems against *L. major* and *L. tropica* using PM sulfate. The results of toxicity assays represented that PM-SLN formulation with smaller size and lower dose was safe with slight toxicity compared to larger size and higher dose of PM-SLN with enhanced effectiveness. Additionally, the results of SYTO green staining and fluorescent microscope imaging showed a decreased number of leishman-bodies in infected THP1 cells treated with PM-SLN. According to the results of in vitro analysis of IC_50_ and EC_50_ of PM-SLN formulation, this formulation was suggested for using in the treatment of the cutaneous leishmaniasis. Furthermore, Heidari‐Kharaji et al. [35], employed PM-SLN formulation against *L. major* in infected BALB/c mice and reported no significant toxicity in laboratory animals. In addition, the levels of cytokines including IL-4, gamma interferon (IFN-γ), and nitric oxide (NO) were evaluated. The results suggested that PM-SLN formulation is effective and safe with great drug delivery properties, which can reduce proliferation of *L. major* parasites in infected mice. In addition, Khosravi et al. [29], developed mannosylated SLNs containing PM (PM-SLN-M) and evaluated the anti-protozoa activity on acute toxoplasmosis. The results proposed high anti intracellular *T. gondii* activity with low cell toxicity in higher dosage than PM (Table 3).

**Table 3:** Current antiparasitic drugs loaded into the SLNs, preparation methods, and characterization techniques

No	Loaded drugs	Parasites	Preparation methods	Characterization methods	References
1	DHA	*P. berghei*	Single-emulsion solvent evaporation/high speed homogenization	XRD SEM PCS	[37]
2	Artemeter/lumefantrine	*P. falciparum*	HPH	PCS CSLM DSC	[38]
3	Tanespimycin 17AAG	*Leishmania* spp.	Double emulsion method/HSH	CLSM TEM	[31]
4	AmB, PM	*L. donovani*	Emulsification solvent evaporation	SEM TEM CLSM	[32]
5	VBS-AmB	*L. donovani*	Double emulsion/solvent-evaporation method/thermal–sensitive hydrogel	FTIR XRD SEM TEM	[33]
6	PM	*L. tropica, L. major*	HSH/microemulsion	Fluorescent microscope	[34]
7	ART	*P. falciparum*	HPH	TEM	[39]
8	PM*	*T. gondii*	Solvent injection method	SEM	[29]
9	PM	*L. major*	HSH	IM	[35]
10	NEO	*T. gondii*	Double emulsifcation method	TEM DLS FTIR	[30]
11	Luteolin/polyethylene glycol (PEG)	*P. falciparum*	Hot and cold Homogenization/hot-microemulsion ultrasonic	HPLC	[40]
12	CS-AmB	*L. donovani*	Solvent emulsification-evaporation	HPLC	[36]

ART, Artemether; DHA, Dihydroartemisinin; PM, Paromomycin; AmB, Amphotericin B; HSH, High shear homogenization; HPH, High pressure homogenization. IM, Inverted microscope; EDX, Energy dispersive X-ray spectroscopy; XRD, X-ray diffraction; FESEM, Field emission scanning electron microscopy; TEM, Transmission electron microscopy; SEM, Scanning electron microscope; VSM, Vibrating sample magnetometer; FTIR, Fourier-transform infrared spectroscopy; DLS, Dynamic light scattering; PDI, Particle size and polydispersity index; EE, Entrapment efficiency; DSC Differential scanning calorimetry; TGA, Thermogravimetric analysis; PS, The mean particle size; ZP, Zeta potential; PCS, Photon correlation spectroscopy; HPLC, High performance liquid chromatography; AFM, Atomic force microscope; CM, Compound; FM, fluorescent microscope; OM, Optical microscopic

^*^ This study used mannosylated SLNs

### Helminths

#### Classification of anthelmintic drugs based on the mechanism of action

Helminths are multicellular microorganisms with a complex body structure and organ systems (e.g., muscular, nervous, digestive, and reproductive). They can involve the liver, blood, intestine, and other tissues in human hosts [156]. From the clinical point of view, helminths are phylogenetically divided into three classes: cestodes or tapeworms, nematodes or roundworms, and trematodes or flatworms, with worldwide distribution [157].

Anthelminthic drugs destruct cell structure, integrity, metabolism, and neuromuscular coordination, which result in damage and the expulsion of worm from host intestine [158, 159]. Anthelminthic agents are generally classified into three groups: anticestodal, antinematodal, and antitrematodal drugs. Anthelmintic drugs may interfere with the carbohydrate metabolism, inhibit respiratory enzymes, and block neuromuscular action of helminths, and make them susceptible to the host's immune cells [160, 161]. Anthelminthic agents are generally classified into three groups: anticestodal, antinematodal, antitrematodal drugs.

##### Disrupting the metabolism

Benzimidazoles (BZD) such as albendazol (ABZ), mebendazol, thiabendazol, and tricolabendazol are a group of therapeutic agents, employed for a broad spectrum of helminths (*E. vermicularis* (pinworm), *T. trichiura* (whipworm), *A. lumbricoides* (common roundworm), *Ancylostoma duodenale* (common hookworm), *Necator americanus* (American hookworm), which mainly inhibit tubulin polymerization [162]. Benzimidazoles acts by interfering with carbohydrate metabolism and inhibiting polymerization of microtubules, which led to damage of cytoplasmic microtubules and impaired uptake of glucose via the larval and adult stages of the parasites [163].

Mebendazole inhibits glucose absorption in nematodes and cestodes, resulting in a significant consumption of parasite glycogen [164]. Niclosamide is an anthelmintic drug which is used to treat tapeworms and inhibits coupling of oxidative phosphorylation reactions and the electron transport, which impairs ATP synthesis [165]. Clorsulon is a compound belonging to the benzenesulphonamide family, which is recommended for treatment and control of adult liver flukes, *F. hepatica* [166]. Clorsulon affects the glycolytic pathway and nervous system of the parasite, resulting in inhibition of phosphoglycerate kinase [167]. Bithionol is an antibacterial, anthelmintic, and anti-algae agent, which had been practiced for treatment of fascioliasis and paragonimiasis before commercializing praziquantel. Bithionol interferes in ATP production and oxidative phosphorylation, which prevents formation of ATP in parasites [164].

##### Frustrating the nervous and muscular system

Imidazothiazoles are a group of anthelminthic drugs, which target neurotransmitters. Imidazothiazoles are nicotinic anthelmintic that act as acetylcholine receptors of nematodes and lead to flaccid paralysis of helminth via neuromuscular depolarizing blockade through stimulation of ganglion-like structures in nematodes muscle cells, and expelling by the normal peristaltic movements of the host intestine [168]. Piperazine is a common drug against nematodes, which through gamma-aminobutyric acid mimicking (GABA) affects chlorine (Cl) channels. The activity of piperazine depends on the inhibition of neuromuscular transmission (succinate production) in the parasite, hyperpolarizing of the nerve membrane, and paralysis of the helminths [169]. Some anthelmintic drugs act rapidly and selectively on neuromuscular transmission of nematodes. Levamisole, pyrantel, and morantel target nicotinic acetylcholine receptors of nematode muscle and cause spastic paralysis [170]. These drugs increase the flow of cations that leads to a rigid paralysis [171]. Pyrantel pamoate has been known as a broad-spectrum anthelmintic drug, which is prescribed for ascariasis, enterobiasis, and hookworm infections. This drug is a depolarizing neuromuscular-blocking agent, which paralyzes helminths by causing acetylcholine release and inhibiting cholinesterase [159].

##### Cell and membrane integrity destruction

Some of anthelmintic drugs destruct the integrity of protective layers and paralyze helminths with leakage of intracellular Ca^2+^. For example, praziquantel (PZQ) leads to tegumental impair and paralytic muscular of cestodes, resulting in death and deportation [172]. PZQ is broadly prescribed for hydatid cyst, neurocysticercosis, clonorchiosis, opisthorchosis, and schistosomiasis [173]. Following treatment with PZQ, tegument is disrupted due to significant influx of Ca^2+^ in the worms and increasing the calcium uptake of the parasite [174]. Diethylcarbamazine is a drug of choice against filiariasis, loiasis, and tropical eosinophilia, which immobilizes microfilariae and alters surface structure of the parasites*.* Furthermore, this drug displaces microfilariae from tissues to increase the contact between the parasites and immune system (Table 4).

**Table 4:** Traditional anthelminthic drugs, the mechanism actions, and side effects

Drugs	Affected genera	Mechanisms of action	Side effects
Albendazole	*T. solium*, *Echinococcus* spp.*, A. lumbricoides,* Hookworms*, Trichostrongylus* spp.*, C. philippinensis, T. trichuris, E. vermicularis, C. sinensis, Gnathostoma* spp.	Inhibiting microtubule synthesis Blocking glucose uptake causing deplete the glycogen stores	Stomach pain Nausea Dizziness thinning or loss of the hair Dark urine Diarrhea Drug resistance of mebendazole to treat *N. americanus*
Praziquantel	*S. mansoni, C. sinensis, O. viverrini, T. solium, H. nana*	Increasing Ca^+2^ uptake Muscle paralysis	Headache Dizziness Stomach pain Nausea Vomiting Tiredness
Diethylcarbamazine	*W. bancrofti, B. malayi, B. timori, Loa Loa*	Interfering with different metabolic processes such as: nitric-oxide synthase and the cyclooxygenase pathway Inhibitor of arachidonic acid metabolism Altering surface structure	Itching and swelling of the face Especially the eyes Headache Joint pain Unusual tiredness or weakness
Triclabendazole	*F. hepatica, F. gigantica*	Inhibiting microtubule synthesis Blocking glucose uptake causing deplete the glycogen stores	Dizziness or lightheadedness Feeling of constant movement of self or surroundings Sensation of spinning Decreased appetite Diarrhea Headache Increased sweating Skin rash Stomach pain
Mebendazole	*E. vermicularis, A. lumbricoides, T. trichiura,* *A. duodenale, N. americanus, M. perstans, T. canis, T. cati*	Inhibiting microtubule synthesis Blocking glucose uptake causing deplete the glycogen stores	Loss of appetite Abdominal Pain Diarrhea Flatulence Nausea Vomiting Headache Tinnitus Elevate liver enzymes
Pyrantel pamoate	*A. lumbricoides, E. vermicularis, M. moniliformis*	Disturbing the function of the neuromuscular apparatus in parasite	Nausea Vomiting Diarrhea Dizziness Poor efficacy of pyrantel pamoate against *A. duodenale*
Ivermectin	*S. stercoralis, O. volvulus, M. ozzardi, M. streptocera, G. spinigerum*	Target GABA receptors of parasite Chloride ion influx Hyperpolarization of parasite muscles and paralyzing	Difficulty in moving Muscle pain or stiffness Pain in the joints Swollen, painful, or tender lymph glands in the armpit Black, tarry stools Chest pain Chills Cold sweats Resistance of *H. contortus* in sheep and goats
Piperazine	*A. lumbricoides, E. vermicularis*	GABA receptor agonist Hyperpolarization of parasite muscles and paralyzing	Blurring of vision Clumsiness Crawling or tingling feeling of the skin Fever Irregular, twisting movement, especially of the face, arms, and legs Joint pain skin rash or itching
Niclozamide	*T. saginata, T. solium, D. latum*	Inhibiting ATP generation	Abdominal or stomach cramps or pain Diarrhea Loss of appetite Nausea or vomiting

#### The therapeutic efficacy of anthelmintic drugs loaded into SLNs

Several classes of anthelminthic agents have been developed, so far. Some drugs are specifically prescribed for a class of helminths. For example, drug of choice for schistosomiasis and tapeworms is praziquantel, while mebendazole and ABZ are the first line drugs prescribed for soil-transmitted helminths, and diethylcarbamazine and ivermectin are prescribed for filarial infections [175].

The site of absorption for anti-helminthic drugs in gastrointestinal tract is different [176]. It was evidenced that mebendazole is accumulated in the intestine and is used for treating large intestinal roundworms such as Ascarids, hookworms, and whipworms [177]. Although most of the drugs are absorbed from the intestinal tract, pyrantel pamoate, a drug of choice for pinworm infection, ascariasis, hookworm infection, and trichostrongilosis, is accumulated in the intestinal lumen [159]. Shortcomings of current anthelmintic drugs are poor water-solubility, low bioavailability, rapid degradation, and clearance factor [178]. Therefore, nanomedicine and conventional therapy have been incorporated for target delivery and reducing anthelminthic resistant [179, 180]. Nanoparticles with high surface to volume ratio provide higher surface area, resulting in reducing the size, increasing the surface area, as well as increase in the dissolution rate of the particles [181]. In addition, employing suitable carrier can overcome the solubility and bioavailability limitations of conventional drugs. It is supposed that gastrointestinal helminths use lipid droplets from digestive fluid to utalize it in their methabolic activity and produce energy [103]. This fact makes SLNs as an excellent lipid carrier for the delivery of anthelmintic drug agents such as: ABZ [99, 103, 105, 182], PZQ [101, 102, 183], and albendazole sulfoxide (ABZS) [99, 100, 104].

Ivermectin and nitazoxanide (NTZ) are effective anthelminthic drugs, which are widely employed for trichinosis [148]. However, drug resistant is expected for these drugs in the future, which arises the emergency for developing new and stable biocompatible therapeutic agents. In this regard, Hassan et al. [148] developed SLNs-loaded nitazoxanide (NTZ-SLNs) to increase the capture efficacy and drug release rates using modified thin film hydration technique for treatment of intestinal and muscular phase of trichinosis in murine host. The histopathological assessments demonstrated that ivermectin only affected the muscular phase, while NTZ-SLNs significantly killed *Trichinella* larvae in both intestinal and muscular phases, indicating increased efficacy of the drugs after combination with SLNs [148]. In addition, thin-film hydration is one of the most commonly used methods for liposome preparation [184]. ABZ is a benzimidazole, which has been widely prescribed for parasitic infections. The efficacy of ABZ has been evaluated against ascariasis, trichuriasis, strongyloidiasis, enterobiasis, and hookworm infection. The mechanism of action of ABZ is suggested to be via decreasing ATP formation and blocking glucose uptake in different stages of helminth’s life cycle. One of the significant advantages of ABZ, is the solubility of this drug in lipid formulation that leads to improve bioavailability and suitable loading capacity of the drug in the SLNs [103]. Sharma et al. [103] developed a ABZ-loaded SLNs (SLN-A) formulation against the intestinal parasitic worm, *Haemonchus contortus,* to enhance the effectivness of ABZ based on evaluation of the motility and mobility assay (AMMA) of adult helminths. In this study, double emulsion technique was used to produce SLNs. This formulation was developed to reduce the needed dosage and side effects of the drug. Dispersion ultrasonic emulsion and nano-emulsion methods were used for preparation of drug-loaded SLNs and drug-free SLNs [103]. Hydatidosis or hydatid disease is caused by the larval stages of cestode (tapeworm) of the genus *Echinococcus* with serious public health problems. Benzimidazole derivatives (albendazole, thiabendazole, and mebendazole) are the currently administrated drug of choice for echinococcosis. Among BZDs, ABZ, as a lipophilic anthelmintic drug, is an effective drug against cystic echinococcosis (CE). However, the conventional drugs have shown systemic side effects. In this regard, a study was designed on ABZ and ABZ sulfoxide (SO)-loaded SLNs formulation to increase drug permeation across the hydatid cyst membrane [100]. Micro emulsification and high shear homogenization methods were used to prepare SLNs. The particle size of prepared formulations showed < 180 nm, and ABZ-SLNs and ABZSO-SLNs showed fast release, and higher permeability and efficacy compared to conventional drug. Hydatid cysts are classified as fertile and infertile cysts. Actually, fertile cysts contain the germinal layer with the brood capsules and protoscoleces, but the germinal layer of infertile cysts is without protoscoleces. Therefore, diffusion of drugs into the cyst layers may led to increased drugs efficacy [185]. Rafiei et al. [99], investigated ultrastructural changes of ABZ and ABZSO and their SLNs-loaded in fertile and infertile hydatid cysts. They micro-emulsification and high shear homogenization methods were engaged to produce lipid nanoparticles. The findings showed higher efficacy of ABZ and ABZSO SLNs-loaded in fertile and small cysts [99]. Toxocariasis is caused by *T. canis* and *T. catis,* causative agents of visceral larva migrants (VLM) and ocular toxocariasis (OT). ABZ is one of the choice drug in treatment toxocariasis, as well. In this regard, Kudtarkar et al. [105], engaged lipid nanoparticles to prepare SLNs using phase inversion temperature method. The mean particle size of ABZ-SLN was ranged of 116 ± 3.51 nm to 168.3 ± 3.92 nm. The anthelmintic efficacy of ABZ-SLN was evaluated in mice infected with *T. canis* larvae. The ABZ-SLN formulations showed effective drug delivery and therapeutic effect at both low (50 mg/kg) and high (100 mg/kg). Furthermore, the results showed, reduction in larvae count in vital organs of mice. Abedi et al. [182], designed a magnetic SLNs-loaded ABZ as an antiparasitic drug. They modified the drug delivery system area in ABZ-SLN formulation. ABZ-magnetic SLNs (MSLNPs) were composed of (Fe_3_O_4_) NPs as the core and stearic acid as shell, were engaged as carriers to increase the efficient delivery of ABZ.

Although the limitations such as poor solubility in water, low absorption through the gastrointestinal tract (GIT), and need for large dosage have been reported [186], PZQ is a frontline drug for parasitic infections and the drug of choice for the schistosomiasis chemotherapy [187]. However, chemotherapy with PZQ is not effective in high endemic areas [186], and the efficiancy of PZQ is low during oral administration. Therefore, nanoformulation of this drug was practiced to overcome these limitations. Souza et al. [98], evaluated the intestinal permeability, toxicity, and effectiveness of PZQ-SLNs against adult *S. mansoni*. The preparation method of SLNs was high-shear homogenization and micro-emulsification. *S. mansoni* was cultured in HepG2 cell line and the adult worms were incubated with SLNs, PZQ-SLNs and free PZQ. PZQ-SLNs were typically spherical shape with a size range between 500 and 1000 nm, and PZQ-loaded SLNs formulation killed the parasites in short time with low cell toxicity against HepG2 cells compared to the free PZQ. Therefore, the PZQ-loaded SLNs were suggested as a promising treatment strategy for the schistosomiasis control program. Similar to previous study, Radwan et al. [97], fabricated a novel PZQ-SLNs formulation to enhance bioavailability and antischistosomal efficacy of PZQ against murine *S. mansoni* infection [97]. In this study, SLN formulations were prepared by micro emulsification and high shear homogenization techniques and the particle sizes of SLNs ranged between 87.32 and 302.3 nm. As results, the number of worms were significantly reduced at 14 days after treatment with SLNs-PZQ in *S. mansoni*-infected mice treated with SLNs-PZQ compared to control group. Moreover, a significant reduction in the percentages of total mature eggs was observed after SLNs-PZQ treatment. The results of pharmacokinetic assessment of PZQ and SLNs-PZQ in normal and *S. mansoni*-infected mice showed enhanced PZQ bioavailability in the *S. mansoni*-infected groups, as well. Xie, et al. [102], developed a modification with loading PZQ into hydrogenated castor oil-SLNs in order to enhance the therapeutic efficacy of the drug against dogs infected with *E. granulosus*. The nanoparticle size was 263.00 ± 11.15 nm, and the preparation methods were hot homogenization and ultrasonication techniques. The hydrogenated castor oil (HCO) was loaded into SLNs to induce the therapeutic efficacy of PZQ. In this study, the *E.* *granulosus*-infected dogs were treated with a low dose of 0.5 mg/kg compared to the high dose of 5 mg/kg, which is prescribed in clinical application, revealing an enhanced pharmacological activity of PZQ by SLNs. Andrade et al. [101] developed SLNs via high-cut homogenization method for the loading of PZQ for the treatment of *S. mansoni* infections. Analysis of SEM microscopy showed that PZQ-loaded SLNs were typically spherical with a size range between 500 and 1000 nm. The in vitro investigation on adult worms of *S. mansoni* showed that PZQ-loaded SLNs has great parasiticidal properties (Table 5).

**Table 5:** The SLNs loaded into current anthelmenthic drugs, Preparation methods of SLNs and characterization techniques are used in parasites

No	Loaded drugs	Parasites	Preparation methods	Characterization methods	References
1	ABZ/Rh-B	*H. contortus*	Double emulsion technique	SEM FTIR Cm FM	[103]
2	ABZ-loaded MSLNPs/ABZ-SLNPs	–	Emulsification dispersion-ultrasonic/Nano-emulsion	XRD EDX TEM VSM FTIR DLS FESEM	[182]
3	PZQ	*S. mansoni*	HSD hot	SEM	[101]
4	PZQ-HCO	*E. granulosus*	Hot homogenization/Ultra-sonication method	SEM PCS HPLC	[102]
5	ABZSO	*E. granulosus*	Solvent diffusion–evaporation	HPLC TEM	[104]
6	ABZSO/ABZ	*E. granulosus*	Micro emulsification/HSD	HPLC AFM	[100]
7	ABZ	*T. canis*	Phase inversion temperature method	XRD DSC TEM	[105]
8	HCO	–	Hot nanoemulsion	HPLC	[188]
9	ABZ/ABZSO	*E. granulosus*	Micro emulsification/ HSD	TEM OM	[99]
10	PZQ	–	Hot homogenization/ Ultra-sonication	TEM DSC XRD	[183]
11	PZQ	–	Oil in-water hot microemulsion/Evaporation of the organic solvent	DSC TGA PCS	[189]
11	NTZ	*T. spiralis*	HSD	UV–vis scan SEM DLS	[148]
12	PZQ	*S. mansoni*	HSD/Micro-emulsification	–	[98]
13	PZQ	*S. mansoni*	HSD/Micro-emulsification	PDI, EE, ZP	[97]

ABZ, Albendazole; NTZ, Nitazoxanide; PZQ, Praziquantel; PRZ, Piperazine; ABZSO, ABZ sulfoxide; MSLNPs, Magnetic solid lipid NPs; HCO, Hydrogenated castor oil; Rh-B, Rhodamine B; HSH, High shear homogenization; HPH, High pressure homogenization; HSD, High shear dispersion; IM, Inverted microscope; EDX, Energy dispersive X-ray spectroscopy; XRD, X-ray diffraction; FESEM, Field emission scanning electron microscopy; TEM, Transmission electron microscopy; SEM, Scanning electron microscope; VSM, Vibrating sample magnetometer; FTIR, Fourier-transform infrared spectroscopy; DLS, Dynamic light scattering; PDI, Particle size and polydispersity index; EE, Entrapment efficiency; DSC Differential scanning calorimetry; TGA, Thermogravimetric analysis; PS, The mean particle size; ZP, Zeta potential; PCS, Photon correlation spectroscopy; HPLC, High performance liquid chromatography; AFM Atomic force microscope; CM, Compound; FM, fluorescent microscope; OM, Optical microscopic

## Future perspective

Together with emerging drugs for a broad spectrum of communicable and non-communicable diseases, nanoscience and nanotechnology are developed. Nanoparticles or nanoscale materials are popular due to their applications in drug delivery, which have led to a dramatically increase in the number of clinical trials of nanomedicine incorporated drugs. Actually, specific drug delivery reduces systemic toxicity and nonspecific distribution, and overcomes limitations of conventional drugs. Despite recent progress in drug delivery systems based on nanomaterials in the treatment of parasites, this subject still remains as a priority of research. However, few clinical trials employed SLN-based formulations as a vehicle for the treatment of the parasite infections, therefore, there is an essential requirement for expanding nanodrugs for parasitic diseases. In addition, together with advances in herbal medicine, SLNs formulation seems to be an alternative technique to either improve specific delivery or decrease toxic effects of herbal extracts or their components.

Application of SLNs formulation in vaccines seems to be another target in future research. It was suggested that incorporation of SLNs with adjuvants can improve the immune responses during vaccination. Importantly, SLNs increase contact surface and control the release of loaded contents. Therefore, regarding the rapid degradation of adjuvants in the body, a combination of the lipid components of SLNs with adjuvants may deliberate degradation of adjutant with long lasting exposure to the immune system, which lead to more efficient immunogenicity.

Although nanotechnology and its applications in nanodrug formulation is rapidly being progressed, the gap between developed and less-developed countries is widening. The lack of resources and technologies in less-income countries decreases the capability of these regions to effectually participate in designing nanotechnology-based drugs for their needs. Apart from the lack of infrastructures in poor countries, the target for effective therapy is also different. The main challenge for less-developed countries is forceful actions against infectious diseases, while the most of developed and commercialized nano-formulated drugs are against non-communicable diseases, which is the main problem in wealth countries. Therefore, the following ways are suggested to increase the chance of designing and prescribing of nanodrugs in less-developed countries. (1) Fabricating cost effective nano-formulated drugs with low maintenance costs, high stability, and high effectiveness to generally decrease the health costs for poor countries. (2) Efficient participation of nongovernment organizations (NGOs) to invest and provide resources for designing and fabrication of effective nanodrugs. (3) The use of low cost materials such as herbal extracts and green technologies in order to decrease the cost for designing novel nanodrugs against infectious diseases. (4) Although there are single studies synthesizing and employing nanodrugs in poor regions, it seems that establishing a headquarter organization to functionally support researches and studies for new technologies, increases the chance of poor regions to access to the nanodrugs and employ them in their challenges.
